# Serotonergic Neurotransmission System Modulator, Vortioxetine, and Dopaminergic D_2_/D_3_ Receptor Agonist, Ropinirole, Attenuate Fibromyalgia-Like Symptoms in Mice

**DOI:** 10.3390/molecules26082398

**Published:** 2021-04-20

**Authors:** Kinga Sałat, Anna Furgała-Wojas

**Affiliations:** Department of Pharmacodynamics, Faculty of Pharmacy, Jagiellonian University Medical College, 9 Medyczna St., 30-688 Krakow, Poland; anna.furgala@student.uj.edu.pl

**Keywords:** reserpine-induced fibromyalgia model, vortioxetine, ropinirole, serotonin and dopamine in fibromyalgia, mouse

## Abstract

Fibromyalgia is a disease characterized by lowered pain threshold, mood disorders, and decreased muscular strength. It results from a complex dysfunction of the nervous system and due to unknown etiology, its diagnosis, treatment, and prevention are a serious challenge for contemporary medicine. Impaired serotonergic and dopaminergic neurotransmission are regarded as key factors contributing to fibromyalgia. The present research assessed the effect of serotonergic and dopaminergic system modulators (vortioxetine and ropinirole, respectively) on the pain threshold, depressive-like behavior, anxiety, and motor functions of mice with fibromyalgia-like symptoms induced by subcutaneous reserpine (0.25 mg/kg). By depleting serotonin and dopamine in the mouse brain, reserpine induced symptoms of human fibromyalgia. Intraperitoneal administration of vortioxetine and ropinirole at the dose of 10 mg/kg alleviated tactile allodynia. At 5 and 10 mg/kg ropinirole showed antidepressant-like properties, while vortioxetine had anxiolytic-like properties. None of these drugs influenced muscle strength but reserpine reduced locomotor activity of mice. Concluding, in the mouse model of fibromyalgia vortioxetine and ropinirole markedly reduced pain. These drugs affected emotional processes of mice in a distinct manner. Hence, these two repurposed drugs should be considered as potential drug candidates for fibromyalgia. The selection of a specific drug should depend on patient’s key symptoms.

## 1. Introduction

According to the American College of Rheumatology, fibromyalgia (FM) is a common neurological health problem that causes widespread musculoskeletal pain accompanied by fatigue, sleep, memory, and mood issues [1,2,3,4]. The development of FM results from a complex dysfunction of the nervous system, and therefore, not only its diagnosis but also treatment and prevention based on combined pharmacological, alternative medicine [5,6], and educational methods [7] are a serious challenge for contemporary medicine [8].

FM affects 2–4% of the population and it is more prevalent in the population of people from urban than rural areas [8,9,10,11,12,13]. Other risk factors comprise female sex, stress, genetic factors, and comorbid inflammatory diseases (e.g., osteoarthritis, rheumatoid arthritis, lupus, and ankylosing spondylitis) [8,14,15,16]. Clinical symptoms of FM arise from the central sensitization [17] due to the neuroendocrine dysfunction and fluctuations in the concentration of neurotransmitters, namely decreased levels of biogenic amines, accompanied by cytokine abnormalities, increased concentrations of excitatory neurotransmitters, and substance P. Impaired functions of the hypothalamic–pituitary–adrenal axis and the autonomic nervous system are also observed in FM patients [18,19].

On the molecular level, the development of FM is based on the impaired serotonin-, noradrenaline-, and dopamine-mediated neurotransmission [20]. This dysregulated neurotransmission is likely to be responsible not only for painful symptoms of FM but also mood control deficits, sleep dysregulation, and cognitive dysfunction as these neurotransmitters serve as neurochemical substrates for a wide range of central nervous system activities [21,22,23].

In view of potential neurochemical mechanisms underlying FM, for years many attempts have been made to search for effective therapies for FM and drugs targeting at serotonergic, noradrenergic, and dopaminergic neurotransmission systems, including serotonin/noradrenaline reuptake inhibitors (duloxetine and desvenlafaxine) [24,25,26,27], or ligands of the α_2_-δ subunit of voltage-gated calcium channels [7,8] have been recommended for FM treatment but their efficacy seems to be modest [25,28]. On the other hand, selective serotonin reuptake inhibitors turned out to be ineffective in the treatment of other than depression symptoms of FM [29].

In the present study we focused on the assessment of two potential drug candidates for FM treatment. The first one affects serotonergic signaling in the brain, while the second one stimulates dopaminergic neurotransmission in the central nervous system. We used a mouse model of FM, i.e., the reserpine (RES) model, and we compared the effects of vortioxetine (VORT), a serotonin transporter (SERT) inhibitor, a 5-HT_3_, 5-HT_7_ receptor antagonist, and a 5-HT_1A_ receptor agonist [30], and ropinirole (ROP), a dopaminergic D_2_ and D_3_ receptor agonist [31], on the pain threshold, depressive-like and anxiety-like responses, locomotor activity and muscle strength of mice exposed to RES to assess if these drugs are able to attenuate the key symptoms resembling those of human FM. The anti-FM effectiveness of multitarget serotonergic agents (e.g., VORT) has not been established, yet. Additionally, the effectiveness of dopamine-mimetics in FM patients requires further confirmation [32] because at present, such compounds are mainly used in the treatment of Parkinson’s disease and restless legs syndrome [33,34]. ROP, because of its previously shown significant impact on decreasing tenderness without causing side effects [35], and pramipexole, which affects muscle pressure and tactile allodynia [36], seem to be promising drugs for FM, even though their therapeutic potential in FM has never been thoroughly investigated.

## 2. Results

**Figure 1:**
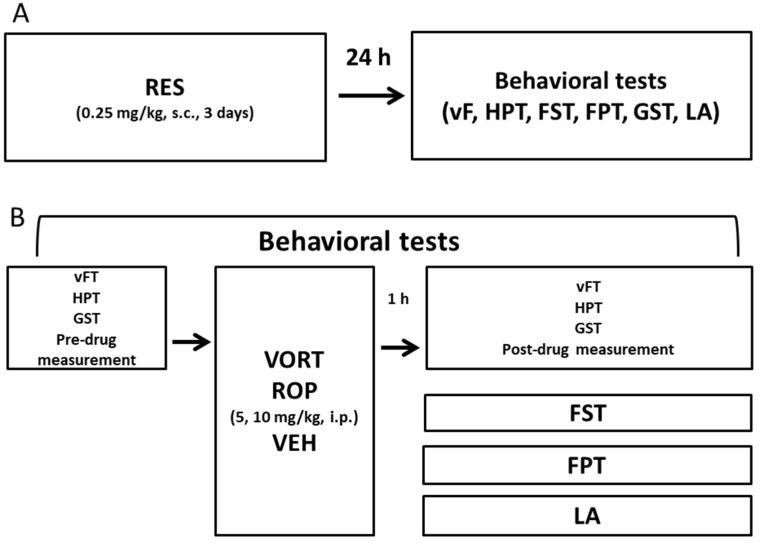
Experimental design used in the present study. FM-like model was induced in mice by a 3-day administration of subcutaneous reserpine (RES; 0.25 mg/kg). Twenty-four hours after the last administration of RES, behavioral tests were carried out (**A**). Vortioxetine (VORT) and ropinirole (ROP) were injected intraperitoneally at doses 5 and 10 mg/kg (**B**). Before test compound administration and 1 h later, the effects of test drugs on tactile allodynia and heat hyperalgesia were measured in the von Frey test (vFT) and the hot plate test (HPT), respectively. Antidepressant-like and anxiolytic-like activities of VORT and ROP were measured in the forced swim test (FST) and the four-plate test (FPT), respectively. Effects of test drugs on the muscle strength were measured using the grip strength test (GST), while the effects of VORT and ROP on the locomotor activity of mice were measured using the locomotor activity test (LA).

The experimental design for in vivo tests is presented in Figure 1. A general procedure used for the induction of FM-like symptoms (Figure 1A) and the behavioral testing protocol used in this study (Figure 1B) are described in Section 4.3 and Section 4.4.

Since this set of experiments, which focused on the assessment of the influence of VORT and ROP on FM-like symptoms, was the first-in-animal study, we investigated pharmacological activities of both drugs at only one time point of testing and we did not carry out time-effect studies. In our study only one route of drug administration was used. Available literature indicates that in pain tests utilizing mechanical and thermal stimulation the maximum effect of oral VORT can be achieved 60–240 min after its administration [37], and oral ROP reaches its peak concentration within approximately 60–120 min [38]. Therefore, in this study we chose 60 min after drug administration as the time point for behavioral testing. We used the intraperitoneal route because the pharmacokinetics of substances administered intraperitoneally resembles that seen after oral administration [39].

### 2.1. Effect on Tactile Allodynia (Von Frey Test)

In the von Frey test repeated-measures ANOVA revealed an overall effect of treatment (F[5, 108] = 62.72, *p* < 0.0001). Time effect and drug × time interaction were also significant (F[1, 108] = 38.25, *p* < 0.0001 and F[5, 108] = 10.98, *p* < 0.0001, respectively).

**Figure 2:**
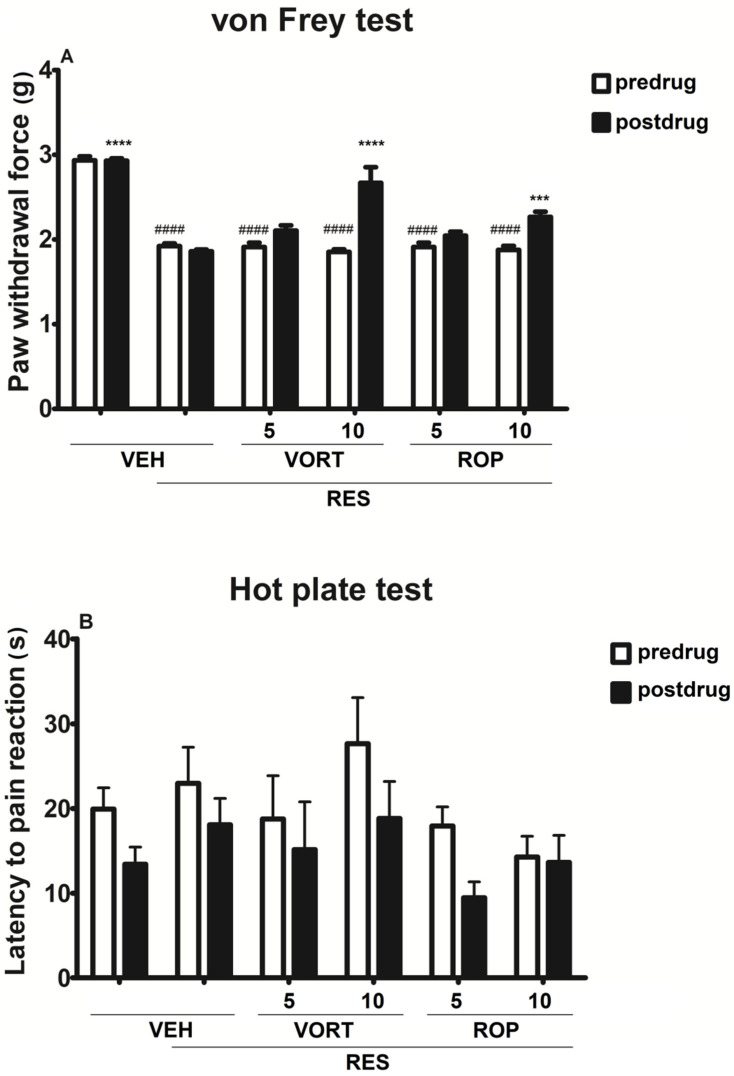
Antiallodynic and antihyperalgesic activities of intraperitoneally administered vortioxetine (VORT; 5 and 10 mg/kg) and ropinirole (ROP; 5 and 10 mg/kg) in the mouse FM-like model induced by a 3-day administration of subcutaneous reserpine (RES; 0.25 mg/kg). The effect of test drugs on tactile allodynia was measured in the von Frey test (**A**) and their effect on heat hyperalgesia was measured in the hot plate test (**B**) 1 h after drug administration. Results are shown as the mean paw withdrawal threshold (g) (**A**), or the mean latency to pain reaction (s) (**B**) ± SEM for *n* = 10. Statistical analysis: repeated measures analysis of variance, followed by Tukey’s post hoc comparison. Significance vs. predrug paw withdrawal of VEH group: #### *p* < 0.0001 and significance vs. postdrug paw withdrawal of RES + VEH group: *** *p* < 0.001, **** *p* < 0.0001.

In this assay (Figure 2A), Tukey’s post hoc analysis showed that RES significantly lowered the mechanical nociceptive threshold in mice (*p* < 0.0001 vs. predrug paw withdrawal of VEH group). VORT and ROP influenced the paw withdrawal threshold of mice treated with RES and compared to RES + VEH group a statistically significant (*p* < 0.001) elevation of the mechanical nociceptive threshold was noted in RES + VORT 10 mg/kg and RES + ROP 10 mg/kg groups. The dose 5 mg/kg of VORT or ROP did not affect the mechanical nociceptive threshold of mice treated with RES (Figure 2A).

### 2.2. Effect on Heat Hyperalgesia (Hot Plate Test)

Repeated measures ANOVA did not reveal an effect of treatment on the hot plate test results (F[5, 108] = 1.994, *p* > 0.05). Time effect was significant (F[1, 108] = 6.455, *p* < 0.05) but the drug × time interaction was not (F[5, 108] = 0.3449, *p* > 0.05). Post hoc analysis did not reveal statistically significant antihyperalgesic properties of VORT or ROP in this assay (Figure 2B).

### 2.3. Effect on Depressive-Like Symptoms (Forced Swim Test; FST)

In the FST one-way ANOVA showed an overall effect of treatment on the duration of immobility in mice (F[5, 51] = 25.00, *p* < 0.0001).

**Figure 3:**
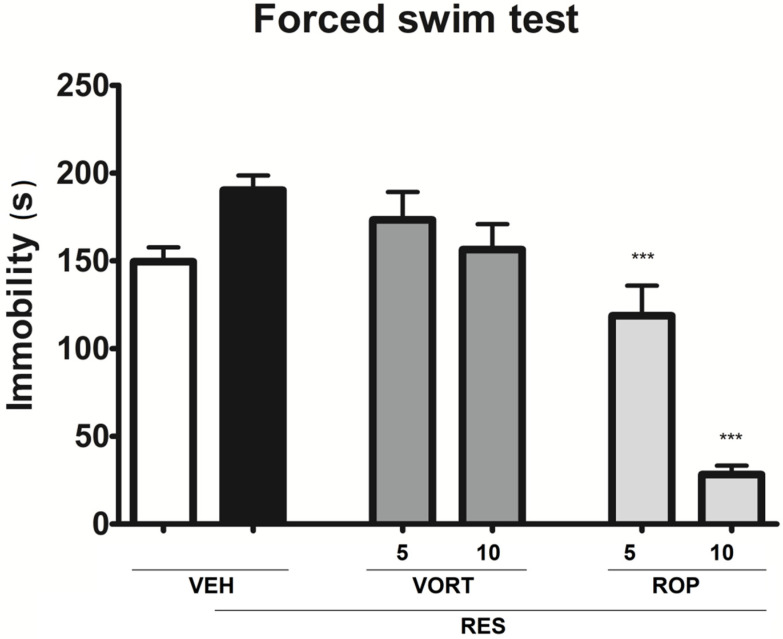
Antidepressant-like properties of intraperitoneally administered vortioxetine (VORT; 5 and 10 mg/kg) and ropinirole (ROP; 5 and 10 mg/kg) in the mouse FM-like model induced by a 3-day administration of subcutaneous reserpine (RES; 0.25 mg/kg). The effect of test drugs on the duration of immobility was measured in the FST carried out 1 h after drug administration. Results are shown as the mean duration of immobility (s) ± SEM for *n* = 8–10. Statistical analysis: one-way analysis of variance, followed by Dunnett’s post hoc test. Significance vs. RES + VEH group: *** *p* < 0.001.

In the FST Dunnett’s post hoc test revealed that although RES prolonged immobility in mice this effect was not specific, i.e., the effect of RES on the duration of immobility compared to that of VEH was not statistically significant (Figure 3). In contrast to this, in mice treated with RES, both doses of ROP significantly reduced the duration of immobility (*p* < 0.001 vs. RES + VEH group; Figure 3). In this test, VORT did not influence immobility of animals, which were previously treated with RES (Figure 3).

### 2.4. Effect on Anxiety-Like Symptoms (Four-Plate Test; FPT)

One-way ANOVA demonstrated a statistically significant overall effect of treatment on the number of punished crossings in the FPT (F[5, 52] = 7.953, *p* < 0.0001).

**Figure 4:**
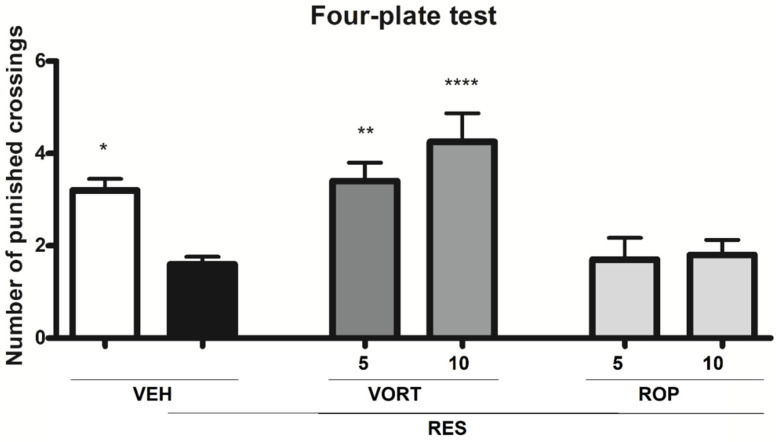
Anxiolytic-like properties of intraperitoneally administered vortioxetine (VORT; 5 and 10 mg/kg) and ropinirole (ROP; 5 and 10 mg/kg) in the mouse FM-like model induced by a 3-day administration of subcutaneous reserpine (RES; 0.25 mg/kg). The effect of test drugs on the number of punished crossings was measured in the FPT performed 1 h after drug administration. Results are shown as the mean number of punished crossings ± SEM for *n* = 8–10. Statistical analysis: one-way analysis of variance, followed by Dunnett’s post hoc test. Significance vs. RES + VEH group: * *p* < 0.05, ** *p* < 0.01, and **** *p* < 0.0001.

Dunnett’s post hoc analysis revealed that the number of punished crossings in the RES + VEH group was significantly lower as compared to the VEH group (*p* < 0.05), RES + VORT 5 mg/kg (*p* < 0.01), and RES + VORT 10 mg/kg groups (*p* < 0.0001, Figure 4). ROP at doses 5 and 10 mg/kg did not show anxiolytic-like properties in the mouse FPT (Figure 4).

### 2.5. Effect on Muscle Strength (Grip Strength Test)

**Figure 5:**
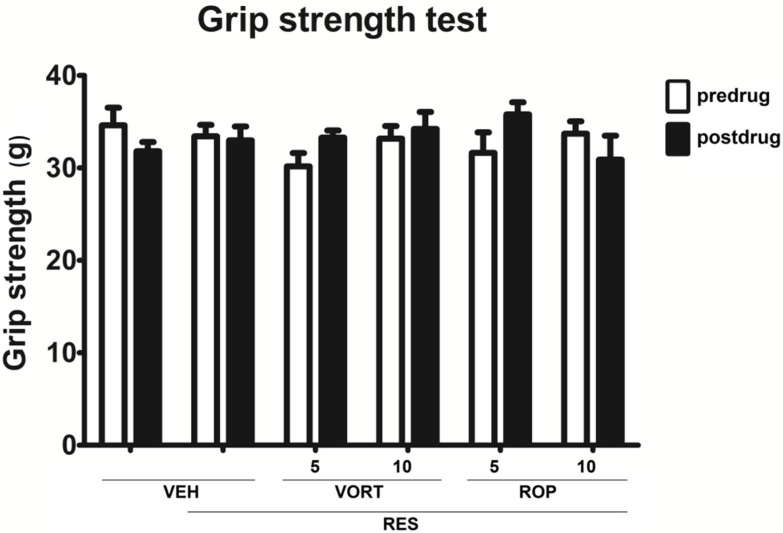
Effect of intraperitoneally administered vortioxetine (VORT; 5 and 10 mg/kg) and ropinirole (ROP; 5 and 10 mg/kg) on the muscle strength in the mouse FM-like model induced by a 3-day administration of subcutaneous reserpine (RES; 0.25 mg/kg). The effect of VORT and ROP was measured in the grip strength test carried out 1 h after drug administration. Results are shown as the mean force (expressed in (g)) that evoked the animal’s reaction ± SEM for *n* = 10. Statistical analysis: repeated-measures analysis of variance, followed by Tukey’s post hoc comparison. Significance vs. RES + VEH group at the respective time point of testing: *p* > 0.05.

The grip strength test was carried out to assess if RES, VORT, or ROP affect muscle strength of mice. Repeated measures ANOVA showed that neither drug effect, time effect, nor the drug × time interaction were significant (F[5, 108] = 0.4860, *p* > 0.05, F[1, 108] = 0.1589, *p* > 0.05, and F[5, 108] = 1.639, *p* > 0.05. respectively) and none of the drugs used affected muscle strength of mice (Figure 5).

### 2.6. Effect on Locomotor Activity

Repeated measures ANOVA demonstrated an overall effect of treatment on the locomotor activity of mice (F[3, 84] = 9.104, *p* < 0.0001). Time effect was also statistically significant (F[2, 84] = 78.90, *p* < 0.0001) but the drug × time interaction was not (F[6, 84] = 1.421, *p* > 0.05).

**Figure 6:**
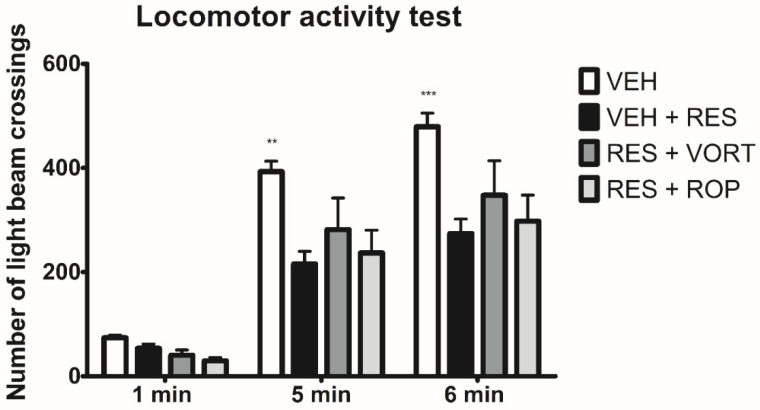
Effect of intraperitoneally administered vortioxetine (VORT; 10 mg/kg) and ropinirole (ROP; 10 mg/kg) on the locomotor activity of mice in the FM-like model induced by a 3-day administration of subcutaneous reserpine (RES; 0.25 mg/kg). The effect of test drugs on the number of light beam crossings was measured in the locomotor activity test carried out 1 h after drug administration. Results are shown as the mean number of light beam crossings ± SEM for *n* = 8. Statistical analysis: repeated-measures analysis of variance, followed by Tukey’s post hoc comparison. Significance vs. RES + VEH group at the respective time point of testing: ** *p* < 0.01 and *** *p* < 0.001.

Locomotor activity did not differ in RES + VEH group compared to RES + VORT group or RES + ROP group. However, locomotor activity of RES + VEH group was significantly decreased as compared to the VEH group (*p* < 0.01 in the 5th min of the test, and *p* < 0.001 in the 6th min of the test, Figure 6).

## 3. Discussion

FM is a disease that results from the impaired neurotransmission mediated by serotonin and catecholamines [40,41,42]. This altered neurotransmission underlies many symptoms of this disease, including chronic pain, mood and sleep disorders, and cognitive decline [43]. In FM dysregulated dopaminergic neurotransmission was shown and a strong correlation between dopamine metabolism and reduced gray matter density was reported. These phenomena contributed to the enhanced pain perception, cognitive deficits and abnormal stress reactivity [44]. Additionally, a disruption of presynaptic dopaminergic neurotransmission in those brain regions where dopamine plays a putative role in analgesia was shown in FM patients [45]. Recent human and animal studies have revealed that a decreased level of dopamine or the hypofunction of the dopaminergic system might lead to a significantly lowered pain threshold [46,47] and chronic pain intensification [48]. In patients suffering from FM, abnormal dopamine function may be associated with differential processing of pain perception [49,50]. It has also been shown that the polymorphism in the serotonergic 5-HT_3_ receptor gene (HTR3) might modulate the striatal dopamine D_2_/D_3_ receptor availability in FM patients [15].

Considering this key role of the abnormal serotonergic and dopaminergic neurotransmission in the development of FM in humans, in the present research we investigated the influence of VORT and ROP on the pain threshold, depressive-like symptoms, anxiety, and motor functions in a rodent model of FM caused by RES.

In this mouse study we used RES, a natural alkaloid [51,52], which acts by inhibiting the sequestration of monoamine neurotransmitters, namely noradrenaline, dopamine, and serotonin into storage vesicles [53,54]. This action triggers increased metabolism of neurotransmitters resulting in their marked decrease in the brain and the spinal cord [55].

Peripheral administration of RES at doses 1–10 mg/kg produces a significant (70–95%) depletion of monoamine content in the central nervous system. This monoamine depletion occurs 30 min after RES injection and may last up to 2 weeks, finally returning to physiological levels after 21 days of retrieval [56].

As proposed by Nagakura and colleagues [36], repeated-dose RES-induced depletion of biogenic amines in the nervous system underlies the development of sensory hypersensitivity accompanied by depressive-like and anxiety-like behavior in rodents [57,58]. Behavioral studies also proved the effect of RES on sleep architecture [59], muscle strength [60], and cognitive impairment [61]. Hence, RES is regarded as a reliable tool compound to induce FM-like symptoms in laboratory animals. It offers good construct validity, face validity, and predictive validity [41] because its use mimics disease biochemistry, many symptoms that occur in FM patients and it is also useful in the search for new biologically active compounds with potential therapeutic effectiveness in FM.

It should be however emphasized that the RES-induced mouse model of FM used in this present study has several limitations, mainly because it is not entirely specific for this disease. Particular attention should be paid to the fact that RES is also used as a tool to model Parkinson’s disease and similarities in behavioral changes in the FM model and the Parkinson’s disease model are observed. As shown by Leal and colleagues [62] repeated administrations of low doses of RES can mimic the progressive nature of Parkinson’s disease. This confirms the ability of RES to produce symptoms similar to those observed in the early stages of this disease, i.e., RES-treated animals show cognitive and emotional deficits in the early stages of this disorder, even before the onset of motor abnormalities. The non-motor symptoms typical for the RES-induced model of Parkinson’s disease have been associated mainly to impairments in the serotonergic and noradrenergic neurotransmission pathways. In this sense, it is not fully understood, whether the model that we used in this present study more closely reflects the symptoms of FM, or rather those which are typical for the early stages of Parkinson’s disease as behavioral changes present in both these disorders can be easily modeled with the use of RES. Due to this issue, future studies using another rodent model of FM should be carried out to confirm the results obtained in the present experiment [62], also considering that at concentrations similar to those used in this present research, RES has still been used in rodents to investigate the pathophysiology of Parkinson’s disease and to demonstrate therapeutic efficacy of drugs and drug candidates for this neurodegenerative disorder [63,64], while its higher dosages varying from 1 to 10 mg/kg induce a wide range of motor impairments that resemble Parkinson’s disease: akinesia, hypokinesia, catalepsy, limb rigidity, and oral tremor. Importantly, some of these motor deficits might influence locomotor activity measures [56,62] and they might be accompanied by memory deficits, anxiety-like behavior, depressive-like behavior, and nociceptive sensitization. Of note, anxiety-like behavior occurs in RES-treated animals in a dose range that does not produce motor impairment (0.1–0.5 mg/kg) and this dose range was used in our study. Moreover, the repeated treatment with low doses of RES (0.1 mg/kg) was suggested as a progressive model of Parkinson’s disease in which motor impairments were preceded by cognitive decline, neuronal alterations compatible with the pathophysiology of Parkinson’s disease, and emotional processing deficits. In this progressive model of Parkinson’s disease, the observed immobility in the FST correlated well with nociceptive sensitization, anxiety, and depression, showing that RES induced non-motor symptoms comorbid with the motor ones [56,65]. Taken together, these data indicate that RES has good face validity for both FM and Parkinson’s disease by inducing symptoms that are noted in both disorders, although for the latter condition, a longer period of RES administration (i.e., 3 weeks) is usually recommended [65]. Additionally, high predictive validity of RES as a tool to assess the efficacy of dopaminergic and non-dopaminergic drugs for both FM and Parkinson’s disease seems to be another potential limitation of our study and the effectiveness of drugs such as ROP in the RES-based Parkinson’s disease model (and FM) must be considered. For ROP increased locomotor activity and the ability to reverse hind limb rigidity were shown previously [64]. In addition to this, ROP at doses almost 2-fold higher than those used in our study was able to reduce catalepsy caused by RES [63].

Chronic and widespread pain is one of the most distressing symptoms of FM that often leads to a significant reduction in the normal physical activity, social, and professional exclusion and it is estimated that the use of both pharmacological and non-pharmacological methods to treat FM symptoms is inadequate in approximately 50% of FM patients [66]. In our present study, RES significantly lowered mechanical nociceptive threshold measured in the von Frey test. It induced anxiety-like symptoms in mice and it significantly reduced animals’ locomotor activity measured in the locomotor activity test. RES also prolonged immobility in the FST, however this effect did not reach statistical significance. It did not affect thermal nociceptive threshold measured in the hot plate test. Muscle strength was not altered by RES, either. Taken together, the analysis of the effects of RES on FM-like symptoms revealed that it mainly influenced the mechanical nociceptive threshold and anxiety-like behavior of mice. Lowered pain threshold for mechanical stimulation [67] and increased anxiety due to RES administration [68] were also shown previously.

In our study we demonstrated that both VORT and ROP used at the dose of 10 mg/kg were able to elevate the mechanical nociceptive threshold and VORT was more efficacious than ROP in this assay. Previous studies revealed analgesic properties of VORT in various pain conditions in mice [37,69] and humans [70,71] and it was suggested that the analgesic properties of VORT in chronic pain conditions were due to the increased content of serotonin and noradrenaline in the brainstem of mice [37]. Our present study is the first one that shows that VORT might also be effective in pain in the course of FM.

In the von Frey test we also demonstrated that ROP, the dopaminergic D_2_/D_3_ receptor agonist was effective as an antiallodynic agent. The role of spinal D_2_ receptor stimulation in the reduction of superficial dorsal horn neuron hyperexcitability has been recently shown [72] and it has been suggested that spinal D_2_ receptors might be promising therapeutic targets for the treatment of pain symptoms in some chronic diseases [72,73]. Interestingly, the latter study used the von Frey test and it showed antiallodynic properties of ROP in a mouse model of Parkinson’s disease. Potential analgesic efficacy of ROP resulting from its agonistic activity at D_2_/D_3_ receptors in FM patients was suggested previously based on results from clinical trials [74,75] and our present study confirmed antiallodynic properties of this drug in a mouse model of this disease.

In contrast to previously published results [55], in our present research RES did not induce heat hyperalgesia in mice. This difference is hard to explain. Specific strain differences or distinct temperature range used during testing might be potential explanation for this discrepancy between these two studies. On the other hand, there are also some studies that showed antinociceptive properties of RES in the hot plate test [76], which might be the explanation for the observed lack of hyperalgesia due to RES administration. In the hot plate test, neither VORT, nor ROP influenced the thermal pain threshold of mice treated with RES.

Acute stress is one of contributing factors to increased depressive symptomatology and anxiety [77]. Stress also plays an important role in the development of FM in humans [16] and for this reason some antidepressants, which act as inhibitors of serotonin and noradrenaline reuptake, were previously found to be effective in FM patients [24,78,79]. The FST is an assay for screening of compounds with potential antidepressant properties and it reveals compounds, even after their acute administration [80]. In this test, increased immobility of mice is not only a measure of depressive-like state but it also shows a coping strategy of a mouse exposed to an acute inescapable stress. Thus, the test provides some insight into the neural mechanisms of the stress response [81].

In our present research RES did not induce depressive-like behavior measured in the FST in mice. Although the 3-day administration of this alkaloid slightly prolonged the duration of immobility, this activity did not reach statistical significance. We can therefore conclude that the FM model based on repeated administrations of RES showed limited face validity for this disorder. Our finding is, however, in contrast with that previously reported by Klein and colleagues [55] who noted a prolongation of immobility of mice injected with RES in the FST.

Interestingly, in the FST we were not able to demonstrate antidepressant-like properties of VORT. In contrast to this, a statistically significant reduction of immobility time was shown for ROP-treated group. The FST results obtained for the antidepressant drug VORT might seem surprising, however the lack of antidepressant-like properties of VORT revealed in this test might be explained as follows: VORT is a drug that acts by modulating serotonergic neurotransmission and according to Cryan and colleagues [82] serotonergic compounds might not reveal their potential antidepressant properties in the classical FST, which was used in the present experiment. In contrast to this, antidepressant-like properties shown for ROP in the FST in a mouse model of FM are in line with the previous reports. They demonstrated that ROP possessed antidepressant-like properties in mice [83,84], which were due to ROP-induced altered functions of dopaminergic, serotonergic, or sigma receptors [83]. This activity of ROP was also confirmed in humans [85].

The above-mentioned previous study [84] showed that ROP significantly and dose- dependently decreased immobility time in experimental animals. Only at the dose of 10 mg/kg it increased swimming and climbing. Different active behaviors are related to different neurotransmitter systems [86]. Specifically, the serotonergic system has been implicated in swimming behavior, while climbing might be related to function of the noradrenergic system. Taken together these data demonstrated that ROP may affect serotoninergic neurotransmission, whereas noradrenergic neurotransmission might be affected only following high doses of this drug. Our present data are in accordance with previous findings, which indicated that ROP decreased duration of immobility time in rodents [83,87]. Those effects of ROP seem to be mediated by D_2_-like receptors, because they were blocked by D_2_-like receptor antagonists (e.g., haloperidole), but not by the D_1_ receptor antagonists (e.g., SCH 23390) [83].

In our present research we also assessed potential anxiolytic-like properties of VORT and ROP in a mouse model of FM induced by RES. In the FPT, RES caused a statistically significant decrease in the number of punished crossings, which was a measure of anxiety-like behavior in this assay [88]. In contrast to the FST, the FPT revealed a significant anxiolytic-like activity of VORT, but not of ROP in mice treated with RES. Anxiolytic properties of VORT were also previously reported [89,90,91,92]. For ROP both anxiety [93,94] and anxiolytic-like properties [84,95] were demonstrated. A possible explanation for the observed lack of anxiolytic-like properties of the D_2_/D_3_ receptor agonist, ROP, in mice with FM-like symptoms shown in our present study might be related to the previously reported correlation between abnormally high D_2_/D_3_ receptor availability in the ventral striatum and enhanced anxiety symptoms in depressed subjects [96]. It should also be noted that an increased dopaminergic neurotransmission involving D_2_ (and D_1_) dopaminergic receptors is postulated as an underlying mechanism for anxiety-like behavior in mice [97].

Pain threshold of mice measured in the von Frey test and the hot plate test, immobility duration assessed in the FST or the number of punished crossings measured in the FPT might be influenced by compounds, which impair locomotor activity or other motor functions of animals used in behavioral tests [88]. This, in turn, can significantly reduce the translational value of the preclinical research. Having this in mind, in order to avoid false positive results in behavioral tests assessing the effect of VORT and ROP on key symptoms of FM, we additionally assessed their effects on the muscle strength and locomotor activity of mice. None of the test drugs altered muscle strength of mice measured in the grip strength test and only RES (but not VORT or ROP) reduced locomotor activity of mice. This finding is in line with previous studies, which showed that RES induced features of akinesia and hind limb rigidity in rats for up to 24 h [56,64]. This effect was dependent on striatal dopamine deficits and it was reversed by dopamine replenishment. In our study, locomotor activity of mice was investigated 24 h after the last RES injection and this might explain the observed decreased locomotor functions in the RES + VEH group. As for intraperitoneal ROP or VORT, we did not demonstrate their effects on the locomotor activity of mice. This is in line with previous studies showing that ROP at doses 1–10 mg/kg showed the antidepressant-like effect in the FST without impairing locomotion in rats [84] or mice [83], and that VORT did not affect locomotor activity of mice [92]. Taken together, it can be concluded that the results obtained for VORT and ROP in pain tests, FST and FPT are not falsified by impaired motor functions of mice treated with these drugs. Additionally, it has to be noted that in mice treated with RES, neither VORT, nor ROP were able to restore motor activity of animals to levels observed in the VEH group.

## 4. Materials and Methods

### 4.1. Animals and Housing Conditions

In vivo experiments were performed at the Department of Pharmacodynamics, Faculty of Pharmacy, Jagiellonian University Medical College, Krakow, Poland. Behavioral tests were carried out between 9 am and 2 pm. All procedures for in vivo tests were performed in full accordance with ethical standards laid down in respective Polish and EU regulations (Directive No. 86/609/EEC and the 1st Local Ethics Committee’s in Krakow approval No. 250/2019). In order to avoid potential bias in data recording the investigators who were involved behavioral assays were blinded to experimental groups.

In behavioral tests adult male CD-1 mice weighing 18–22 g were used. The animals were kept in groups of 10–15 mice in cages at standard laboratory conditions (room temperature of 22 ± 2 °C, light/dark (12:12) cycle, humidity 50% ± 10%, free access to food and water, and environmental enrichment before the experiments). The ambient temperature of the room and humidity were kept consistent throughout all the tests. For all experiments the animals were selected randomly. Each experimental group of mice consisted of 8–10 animals/dose. Immediately after the experiment the animals were euthanized.

### 4.2. Chemicals

RES, VORT, and ROP were purchased from Sigma Aldrich (Poznan, Poland). For in vivo tests, the solution of RES was prepared by dissolving an appropriate amount of this drug in the glacial acetic acid (Polskie Odczynniki Chemiczne, Gliwice, Poland) and diluted to a final concentration of 0.5% acetic acid with phosphate buffer (PBS, BioShop Canada Inc., Burlington, ON, Canada). VORT and ROP were suspended in 1% solution of Tween 80 (Pol-Aura, Zabrze, Poland). Test drugs were administered by the intraperitoneal route at a constant volume of 0.1 mL/10 g, 1 h before behavioral tests. Two fixed doses of VORT and ROP were used in this study. Dose selection was based on the previously published literature data [83,98,99]. Control mice received 1% Tween 80.

### 4.3. Induction of FM-Like Model in Mice

The mouse FM-like model induced by RES administration is based on causing a decrease in the concentration of biogenic amines, mostly dopamine and serotonin in the structures of the central nervous system, resulting in a decreased pain threshold and induction of depressive-like and anxiety-like responses. This model was established by Nagakura and colleagues [36] to be used in rats and then adapted for mice by de Souza and colleagues [57]. To induce symptoms mimicking human FM, mice were injected subcutaneously with RES (0.25 mg/kg) for the three consecutive days. Behavioral tests were carried out 24 h after the last RES administration (Figure 1).

### 4.4. Behavioral Tests

#### 4.4.1. Effect on Pain Threshold

Pain threshold of RES-treated mice was assessed at two time points, i.e., before the test drug (VORT, ROP, or VEH) administration (referred to as ‘predrug’ measurement) and 60 min after its administration (‘postdrug’ measurement). Both mechanical and thermal nociceptive thresholds were assessed in each experimental group using the von Frey test and the hot plate test, respectively.

##### Influence on Tactile Allodynia (Von Frey Test)

The test was carried out according to a procedure previously described [100]. The electronic von Frey unit (Bioseb, Vitrolles, France) is a device supplied with a single flexible filament applying the increasing force (from 0 to 10 g) against the plantar surface of the hind paw of a mouse. In this assay the nocifensive paw withdrawal response automatically turned off the stimulus and the mechanical pressure that evoked the response was recorded. On the day of the experiment, the mice were placed individually in test compartments with a wire mesh bottom and were allowed to habituate for 1 h. After the habituation period, in order to obtain baseline (predrug) values of mechanical nociceptive threshold, each mouse was tested 3 times alternately in each hind paw, allowing at least 30 s between each measurement. Then, the mice received tested compounds or vehicle and 1 h later they were tested again and mean postdrug values were obtained for each mouse.

##### Influence on Thermal Hyperalgesia (Hot Plate Test)

The hot plate test evaluated the effect of compounds on thermally-induced pain. The assay was carried out as previously described [100]. Briefly, the mice were placed on a plate with a surface temperature of 55–56 °C (hot/cold plate analgesiameter, Bioseb, Vitrolles, France). The predrug latency to pain reaction, i.e., the hind paw licking or jumping was measured in all experimental groups. Then, the test compounds were injected and 1 h later the test was carried out again to obtain postdrug measurements. To prevent paw tissue damage, the cut-off time of 60 s was established and mice not responding within 60 s were removed from the apparatus and assigned a score of 60 s.

#### 4.4.2. Assessment of Antidepressant-Like Activity (Forced Swim Test, FST)

The FST was carried out according to the method originally described by Porsolt and colleagues [101] with some modification [102]. Mice were dropped individually into glass cylinders (height: 25 cm, diameter: 10 cm) filled with water (maintained at 23–25 °C) to a height of 10 cm. The whole test lasted for 6 min, during which after an initial 2-min period of vigorous activity, each mouse assumed an immobile posture. The total duration of immobility was recorded during the final 4 min of the whole 6-min testing period and the results obtained were compared between control and drug-treated groups of mice. Animals were regarded to be immobile when they remained floating passively in the water, making only small movements to keep their heads above the water surface.

#### 4.4.3. Assessment of Anxiolytic-Like Activity (Four-Plate Test, FPT)

The FPT test was carried out according to a previously described method [103]. The four-plate apparatus (Bioseb, Vitrolles, France) is a cage (25 cm × 18 cm × 16 cm) that is floored with four identical, rectangular plates (11 cm × 8 cm) separated from each other by 4-mm gaps. An electric stimulus transmitted to plates (0.6 mA, duration: 0.5 s) was produced by an electric shock generator. In this test, after a 15-s habituation period, each animal was subjected to an electric shock when crossing from one plate to another (two limbs on one plate and two on another) with a 3-s break between each shock. The total duration of this test was 60 s. The number of punished crossings were counted and compounds with anxiolytic-like properties were able to increase the number of punished passages.

#### 4.4.4. Assessment of the Effect on Muscle Strength (Grip-Strength Test)

The grip-strength apparatus (TSE Systems, Bad Homburg, Germany) was supplied with a steel wire grid (8 cm × 8 cm) connected to anisometric force transducer. In this assay, each mouse was gently kept by its tail so that it could grasp the grid mounted to a high-precision force sensor of the grip-strength device with its forepaws. Then, the animal was pulled back gently by the tail until it released the grid and the maximal grip strength value (expressed in grams) was recorded for each animal. This procedure was repeated three times and the mean of three measurements for each mouse was calculated [104].

#### 4.4.5. Assessment of the Effect on Locomotor Activity

The locomotor activity test was performed using activity cages (40 cm × 40 cm × 31 cm, Ugo Basile, Varese, Italy) supplied with I.R. beam emitters connected to a counter for the recording of light-beam interrupts [102]. Sixty minutes before the locomotor activity test, the mice were pretreated with VORT, ROP, or VEH. Then, they were individually placed in the activity cages for an adaptation period (30 min). The animals’ locomotor activity (i.e., the number of light beam crossings) was counted during the next 6 min in 1-min intervals.

### 4.5. Data Analysis

Data analysis was performed using GraphPad Prism software (version 8.0, San Diego, CA, USA). Numerical results obtained in behavioral tests are expressed as the mean ± SEM. Statistical analysis was carried out using a one-way analysis of variance (ANOVA), followed by Dunnett’s post hoc comparison to compare results obtained for drug-treated groups and the control group. Repeated measures ANOVA and Tukey’s post hoc comparison were used for group comparisons made repeatedly at different time points. *p* < 0.05 was considered significant.

## 5. Conclusions

To sum up, the treatment of EM is a challenge for contemporary medicine and novel treatment options for this disorder are a serious medical demand. In the present study a mouse model of FM was used to assess if two drugs modulating serotonergic and dopaminergic neurotransmission, namely VORT and ROP, might be potentially useful in the treatment of key symptoms of FM in humans. Both drugs markedly reduced tactile allodynia caused by RES, but they affected emotional processes of mice in a distinct manner. VORT reduced anxiety-related behavior induced by RES in mice, while ROP showed an- tidepressant-like properties in RES-treated mice but these RES-exposed mice did not develop depressive-like phenotype. Hence, we concluded that these two repurposed drugs should be considered as potential drug candidates for FM patients, but the selection of a specific drug (i.e., either VORT or ROP) should depend on a careful analysis of the type of patient’s key symptoms (severe anxiety or depressive state comorbid chronic pain).

The alterations of central nervous system neurotransmitter levels induced by RES used to develop the FM-like animal model, could be translated into psychiatric and neurological impairments typically observed in FM patients. However, it is worth noting that the model that was used in this present study has some limitations, of which the lack of full specificity for FM and the ability of this model to reflect symptoms typical for Parkinson’s disease are of particular concern. Therefore, future studies should be focused on testing VORT and ROP in other FM models, such as the acid saline-induced pain model, sound, intermittent cold, and subchronic swimming stress models. As FM is a diverse syndrome that involves multiple etiologies and multiple subtypes, the combined use of these models may be relevant for the assessment of pathways and mechanisms underlying this disease. These combined models are able to mimic particular biomarkers and clinical conditions observed in FM patients and may contribute to a successful development of drugs and their combinations for FM [105].

Since we demonstrated that the RES model did not show signs of depressive-like behavior measured in the FST, and FST was not sensitive enough to measure this FM symptom, we also proposed using additional methods to study antidepressant-like potential of VORT and ROP. Such behavioral assays (e.g., the novelty-suppressed feeding test) were previously used for the validation of RES model of FM [106] but they also seem to be of interest in terms of their potential usefulness to develop drugs for FM. Other behavioral methods for measuring muscle strength deficits and heat hyperalgesia in mice exposed to RES should also be considered in future studies as the tests we used (grip strength test and hot plate test) did not reveal differences between animals treated and animals not treated with RES.

## Data Availability

The data presented in this study are available on request from the corresponding author.

## Sample Availability

Samples of the compounds tested in this study are available from the authors or can be purchased from the official suppliers.

## Abbreviations

FM	Fibromyalgia
HTR3	Serotonergic 5-HT_3_ receptor gene
HPT	Hot plate test
FST	Forced swim test
FPT	Four-plate test
GST	Grip strength test
LA	Locomotor activity test
SERT	Serotonin transporter
RES	Reserpine
ROP	Ropinirole
VEH	Vehicle
vFT	Von Frey test
VORT	Vortioxetine
